# Transfer Information Energy: A Quantitative Indicator of Information Transfer between Time Series

**DOI:** 10.3390/e20050323

**Published:** 2018-04-27

**Authors:** Angel Caţaron, Răzvan Andonie

**Affiliations:** 1 Department of Electronics and Computers, Transilvania University, Braşov 500024, Romania; 2 Corporate Technology, Siemens SRL, Braşov 500203, Romania; 3 Department of Computer Science, Central Washington University, Ellensburg, WA 98926, USA

**Keywords:** Transfer Entropy, time series prediction, information transfer, information energy, IoT data analysis

## Abstract

We introduce an information-theoretical approach for analyzing information transfer between time series. Rather than using the Transfer Entropy (TE), we define and apply the Transfer Information Energy (TIE), which is based on Onicescu’s Information Energy. Whereas the TE can be used as a measure of the reduction in uncertainty about one time series given another, the TIE may be viewed as a measure of the increase in certainty about one time series given another. We compare the TIE and the TE in two known time series prediction applications. First, we analyze stock market indexes from the Americas, Asia/Pacific and Europe, with the goal to infer the information transfer between them (i.e., how they influence each other). In the second application, we take a bivariate time series of the breath rate and instantaneous heart rate of a sleeping human suffering from sleep apnea, with the goal to determine the information transfer heart → breath vs. breath → heart. In both applications, the computed TE and TIE values are strongly correlated, meaning that the TIE can substitute the TE for such applications, even if they measure symmetric phenomena. The advantage of using the TIE is computational: we can obtain similar results, but faster.

## 1. Introduction

According to Judea Pearl, causal analysis goes one step further than statistical analysis, since it aims to infer not only the likelihood of events under static conditions, but also the dynamics of events under changing conditions [1]. Practically, it is very difficult to establish causality between two correlated events. In contrast, it is relatively easy to establish a statistically significant correlation. Whenever correlation is observed, causality is wrongly inferred and human intuition has evolved such that it has learned to identify causality through correlation. This is because of the inability to detect a time lag between a cause and effect which is a prerequisite for causality [2]. The time lag is essential and, according to Shadish et al., there are three key criteria for inferring a cause and effect relationship: (1) the cause preceded the effect; (2) the cause was related to the effect, and (3) we can find no plausible alternative explanation for the effect other than the cause [3].

Causal analysis is not merely a search for statistical correlations, but an investigation of cause–effect relationships. Although, in general, statistical analysis cannot distinguish genuine causation from spurious co-variation in every conceivable case, this is still possible in many cases [1]. Causality is usually posed using two alternative scenarios: the Granger causality and the information-theoretical approach (based on the Kullback–Leibler divergence or the TE).

The Granger (Clive Granger, recipient of the 2003 Nobel Prize in Economics) causality test [4] is a statistical hypothesis test for determining whether one time series is useful in forecasting another. According to Granger, causality could be reflected by measuring the ability of predicting the future values of a time series using past values of another time series. The Granger test is based on linear regression modeling of stochastic processes. More complex extensions to nonlinear cases exist, but these extensions are more difficult to apply in practice [5].

The TE was introduced by Schreiber [6], not as a causality indicator, but as an information transfer measure used to quantify the statistical coherence between time series. The TE is able to distinguish driving and responding elements and to detect asymmetry in the interaction of time series. For instance, in the financial market, based on the TE concept, Kwon and Oh [7] found that the amount of information transfer from index to stock is larger than from stock to index. It indicates that the market index plays a role of major driving force to individual stock. Later, the TE was related to Granger’s causality. Barnett et al. proved that the Granger causality and the TE causality measure are equivalent for time series which have a Gaussian distribution [8]. Hlaváčková-Schindler generalized this result [9].

Assuming that two time series are both Gaussian and that the relationship between them is linear, then the causal direction between the two time series is unidentifiable [10]. The inability to decide between these two models under the assumptions of linearity and Gaussianity is one of the motivations for the well-known saying that “correlation does not equal causality”. It is possible to change the situation by modifying any of these two assumptions (Gaussianity and linear relationship). One option is to assume that at least one of the series is non-Gaussian. Another modification that makes the causal direction identifiable is to assume a nonlinear relationship [10]. In our approach, we do not make any assumptions about Gaussianity or linear relationship.

Generally, we accept that causality and correlation are different. However, sometimes, information transfer and causal effect are not appropriately distinguished. There are differences between the general concept of causality (not necessarily the Granger causality) and information transfer. Causality is typically related to whether interventions on a source can be identified to have an effect on the target, rather than whether observations of the source can help predict state transitions on the target. This latter concept is called *information transfer*, whereas causality may support information transfer or it may support distributed information storage instead [11]. In general, information transfer refers to a directional signal or communication of dynamic information from a source to a destination.

The body of literature regarding quantification of information transfer appears to subsume two concepts: predictive or computational information transfer, and causal effect or information flow. According to Lizier and Prokopenko [12], they are complementary concepts: Causal information flow describes the causal structure of a system, while information transfer can then be used to describe the emergent computation on that causal structure.

Our contribution is a novel information-theoretical approach for analyzing information transfer between time series. Rather than using the relatively well-known Kullback–Leibler divergence and the TE (both based on a measure of uncertainty—the Shannon entropy), we introduce the TIE, which is based on a measure of certainty—the Onicescu Information Energy (IE) [13]. In the Experimental Section, we apply and compare the TIE and the TE on two applications: one in financial time series and the other one in the study of heart-breath interaction. We have to note that there is a symmetry between the TE and the TIE: the TE measures the reduction in uncertainty about one time series given another, whereas the TIE measures the increase in certainty about one time series given another. We claim that the TIE can substitute the TE for detecting information transfer relationships between time series, with the advantage of being faster to compute.

To avoid possible confusion, we aim to highlight the differences between information flow and causality. Similar to the TE, the TIE is a directional, dynamic measure of predictive information, rather than a measure of the causal information flow from a source and to a destination. Meanwhile, similar to the TE, the TIE is a nonlinear extension of the Granger causality. To be interpreted as information transfer, the TE and the TIE should only be applied to causal information sources for the given destination. In this context, we use the information transfer (measured by the TE and the TIE) to establish the presence of and quantify causal relationships. For a comprehensive discussion, see [12].

This paper is an extended version of a conference paper [14]. It is organized as follows. Section 2 introduces the standard TE notations and lists some of its well-known applications. Section 3 introduces the TIE. The financial application is presented in Section 4 and the heart-breath interaction study in Section 5. The paper is concluded in Section 6.

## 2. Background: Transfer Information Entropy

The recent literature on TE applications is rich. A comprehensive introduction to TE is provided by Bossomaier et al. [11], whereas an overview of causality detection based on information-theoretic approaches in time series analysis can be found in [15]. A non-parametric characterization of causality relying on conditional entropy was proposed by Baghli [16].

TE measures the directionality of a variable with respect to time based on the probability density function (PDF). For two discrete stationary processes *I* and *J*, TE relates *k* previous samples of process *I* and *l* previous samples of process *J* and is defined as follows [6,17]:

(1) $${\mathrm{TE}}_{J\to I}=\sum_{t=1}^{n-1}p\left(i_{t+1},i_{t}^{\left(k\right)},j_{t}^{\left(l\right)}\right)\;log\frac{p(i_{t+1}|i_{t}^{\left(k\right)},j_{t}^{\left(l\right)})}{p(i_{t+1}|i_{t}^{\left(k\right)})},$$

where $i_{t}$ and $j_{t}$ are the discrete states at time *t* of *I* and *J*, respectively; and $i_{t}^{\left(k\right)}$ and $j_{t}^{\left(l\right)}$ are the *k* and *l* dimensional delay vectors of time series *I* and *J*, respectively. The three symbols $i_{t+1},i_{t}^{\left(k\right)},j_{t}^{\left(l\right)}$ for computing probabilities are sequences of time series symbols.

$T_{J\to I}$ measures the extend to which time series *J* influences time series *I*. The TE is asymmetric under the exchange of $i_{t}$ and $j_{t}$, and provides information regarding the direction of interaction between the two time series. In fact, the TE is an equivalent expression for the conditional mutual information [15].

Accurate estimation of entropy-based measures is notoriously difficult and there is no consensus on an optimal way for estimating TE from a dataset [18]. Schreiber proposed the TE using correlation integrals [6]. The histogram estimation approach with fixed partitioning is the most widely used. This method is simple and efficient, but not scalable for more than three scalars. It also has another drawback: it is sensible to the size of bins used. Since estimating the TE reduces to the non-parametric entropy estimation, other entropy estimation methods have been also used for computing the TE [18,19,20]: kernel density estimation methods, nearest-neighbor, Parzen, neural networks, etc.

Applications of TE to date has mainly been concentrated in neuroscience, bioinformatics, artificial life, climate science, finance and economics [11].

## 3. Transfer Information Energy

The TIE was introduced by us [14]. In this section, we aim to review its basic notations. We start by defining the IE, introduced in 1966 by Octav Onicescu as a measure of certainty [13].

The IE of a discrete random variable *I* with possible values $\left\{i_{1},i_{2},\ldots ,i_{n}\right\}$ is the expected value of the information content of *I* [21], $H\left(I\right)=-\sum_{t=1}^{n}p\left(i_{t}\right)\;log\;p\left(i_{t}\right)$, whereas the IE is the expected value of the probabilities of the possible values of *I* , $\mathrm{IE}\left(I\right)=\sum_{t=1}^{n}p\left(i_{t}\right)\cdot p\left(i_{t}\right)$.

In general, any monotonically growing and continuous probability function can be considered as a measure of certainty and the IE is such a function. The IE is a special case of Van der Lubbe et al. certainty measure [22] and was interpreted by several authors as a measure of expected commonness, a measure of average certainty, or as a measure of concentration, and is not related to physical energy.

We define the TIE:

(2) $${\mathrm{TIE}}_{J\to I}=\sum_{t=1}^{n-1}p\left(i_{t+1},i_{t}^{\left(k\right)},j_{t}^{\left(l\right)}\right)\left(p\left(i_{t+1}|i_{t}^{\left(k\right)},j_{t}^{\left(l\right)}\right)-p\left(i_{t+1}|i_{t}^{\left(k\right)}\right)\right),$$

to quantify the increase in certainty (energy) of process *I*, knowing *k* previous samples of process *I* and *l* previous samples of process *J*. Similar to the TE, the TIE is asymmetric and measures relationships between time series *I* and *J*. For computational reasons, we take k=l=1.

Both Equations (1) and (2) can be rewritten substituting the conditional probabilities:

(3) $${\mathrm{TE}}_{J\to I}=\sum_{i_{t+1},i_{t},j_{t}}p\left(i_{t+1},i_{t},j_{t}\right)\log\frac{p\left(i_{t+1},i_{t},j_{t}\right)p\left(i_{t}\right)}{p\left(i_{t+1},i_{t}\right)p\left(i_{t},j_{t}\right)},$$

(4) $${\mathrm{TIE}}_{J\to I}=\sum_{i_{t+1},i_{t},j_{t}}p\left(i_{t+1},i_{t},j_{t}\right)\left(\frac{p(i_{t+1},i_{t},j_{t})}{p(i_{t},j_{t})}-\frac{p(i_{t+1},i_{t})}{p(i_{t})}\right).$$

Comparing Equations (3) and (4), we observe that for TE we have four multiplications/divisions and one logarithm, whereas for TIE we have three multiplications/divisions and one subtraction. Considering all operations equivalent, the TIE is theoretically 20% faster, which is obviously a rough theoretical estimate.

The histogram estimation of TE and TIE between two time series can be computed in three steps: (a) transformation of the continuous valued time series into series with discrete values by binning, which resultd in a sequence of tokens selected from an alphabet with as many symbols as the number of bins; (b) evaluation of the probabilities $p(i_{t+1},i_{t},j_{t})$, $p(i_{t})$, $p(i_{t+1},i_{t})$, and $p(i_{t},j_{t})$, for all $i_{t}$ and $j_{t}$; and (c) computation of TE and TIE by using using Equations (3) and (4).

## 4. Transfer Energy between Financial Time Series

A hot application area of causal relationships is finance. Most investors in the stock market consider various indexes to be important sources of basic information that can be used to analyze and predict the market perspectives. We may be interested in the information transfer between two time series such as a market/bench index and an individual stock/ETF products (an ETF (Exchange Traded Fund) is a marketable security that tracks an index, a commodity, bonds, or a basket of assets such as an index fund). Without intending to be exhaustive, we mention the following two papers which describe time series information transfer analysis with TE. Other recent results can be found in [23,24].

Kwon et al. [25] computed the information transfer between 25 stock markets to determine which market serves as a source of information for global stock indexes. They analyzed the daily time series for the period of 2000–2007 using TE to examine the information transfer between stock markets and identify the hub. They concluded that the American and European markets are strongly clustered and they are able to be regarded as one economic region, while Asia/Pacific markets are economically separated from American and European market cluster. Therefore, they could infer that American and European stock markets fluctuate in tune with a common deriving mechanism. The considerable quantity of the TE from American and European market cluster to the Asia/Pacific markets is the strong evidence that there is an asymmetry of information transfer between the deriving mechanisms.

Sandoval [26] used the stocks of the 197 largest companies in the world, in terms of market capitalization, in the financial area, from 2003 to 2012. He studied the causal relationships between them using TE. He could assess which companies influence others according to sub-areas of the financial sector. He also analyzed the exchange of information between those stocks and the network formed by them based on this measure, verifying that they cluster mainly according to countries of origin, and then by industry and sub-industry.

In this context, using real-world financial data, we aim to determine the information transfer between pairs of time series which represent stock market indexes. We use the TIE and the TE, and then compare the results. This application was first described by us [14].

We illustrate with all details the estimation of TI and TIE on a real-world data set, to make the procedure reproducible.

For 20 stock market indexes from the Americas, Asia/Pacific and Europe (Table 1), we estimate the TE and TIE for all pairs. The working days of the markets across the world may vary from one country to another. Therefore, the time series are aligned by time stamp and the missing values are replaced with the previous available ones. We estimate TE and TIE as follows:

(a) Discretization: Binning the Time Series

We slice the domain limited by the minimum and maximum values from the whole data set into equally sized intervals which are then labeled by assigning a symbol to each of them. The result is a sequence of characters, for which we compute the probabilities needed in Equations (3) and (4).

**Figure 1:**
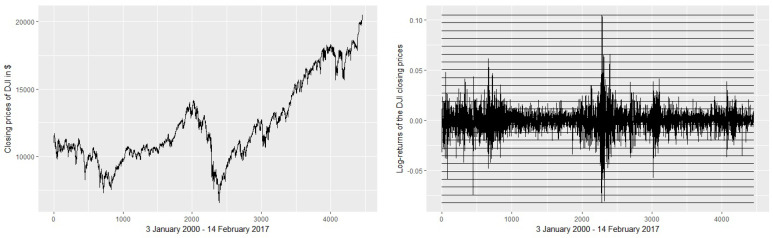
Binning the time series. The left graph presents the raw values of the DJI stock range during 3 January 2000–14 February 2017. On the right side, we represent the log-returns of closing prices and the slicing of the values domain, with 24 equal intervals between minimum and maximum values. Each interval is labeled with a symbol (a letter).

When the binning is applied on the first log-returns of stocks, the narrow bins provide more information content, thus a higher value of entropy *H* than the large bins. Nevertheless, the correlation between the two choices of binning is high in general, reflecting an important similarity of the approaches [26]. In general, for shorter time series, it is advisable to use larger bins to avoid excessive fragmentation (and thus very low or uniform probabilities of symbols). We use 24 bins, noting that the binning strategy is less relevant in our case, since we are not interested in absolute values for TE and TIE, but in their relative values (for comparison). Figure 1 depicts the binning and Table 2 shows a numerical example of binning based on the first values of the DJI and HSI stock indexes.

(b) Compute the Marginal and Joint Probabilities in Equations (3) and (4)

We denote by TE_*t*_ the term under the sum sign in Equation (3) and by TIE_*t*_ the term under the sum sign in Equation (4). The next step is to evaluate TE_*t*_ and TIE_*t*_ by counting the number of each occurrence (Table 2). The string obtained by binning the log-returns of the DJI stock starts with the symbols “g l m n l j k k m k …”. Therefore, $p(i_{1})=0.00673$ is the probability of occurrence of symbol “g”, $p(i_{2})=0.21054$ is the probability of occurrence of symbol “l”, etc. The probability $p(i_{2},i_{1})=0.00179$ is the probability of the sequence “gl”, $p(i_{3},i_{2})=0.00942$ is the probability of “lm”, etc. The string obtained by binning the log-returns of the HSI stock starts with the symbols “k f i n o m l l l m …”. We obtain the probability of “gk”: $p(i_{1},j_{1})=0.00224$; the probability of “gk”: $p(i_{2},j_{2})=0.00224$; etc. Next, $p(i_{2},i_{1},j_{1})=0.00067$ is the probability of “lgk”, $p(i_{3},i_{2},j_{2})=0.00022$ is the probability of “mlf”, etc. For an accurate estimation, a larger number of decimals is preferred.

(c) Estimate TE and TIE

We calculate TE_*t*_ and TIE_*t*_. For the first step, TE_1_ = 0.000011 nn TIE_1_ = 0.0000022, etc. Finally, we compute TE = 47.76 and TIE = 17.85, summing-up the partial results.

**Figure 2:**
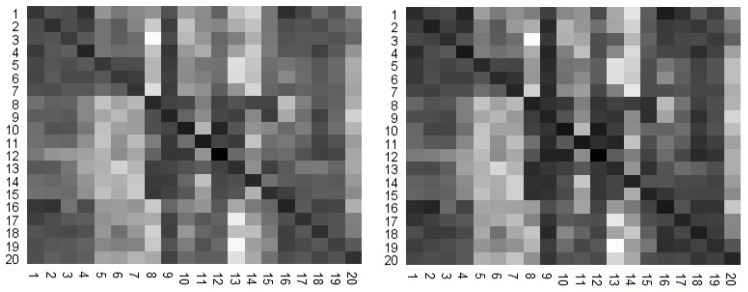
Two heat maps are calculated for TE (**left**) and TIE (**right**), between all combinations of the 20 stock indexes.

The results are summarized in heat maps (Figure 2). The lighter shaded pixels are associated with a higher values of TE and TIE. We visually observe that the two heat maps correlate well. In fact, Pearson correlation coefficient is 0.973, showing a strong correlation.

**Figure 3:**
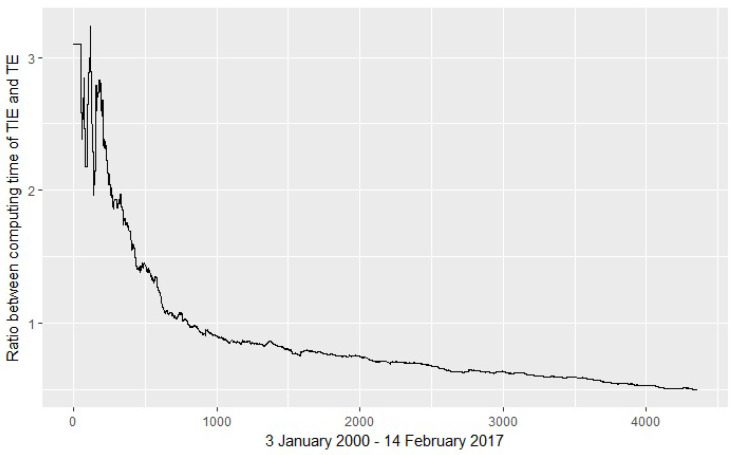
Execution time. The graph shows the ratio TIE/TE of the executions times, for an increasing number of values. The time is computed for the DJI and HSI stocks ranges during 3 January 2000–14 February 2017. The relative efficiency of TIE increases for larger time series. For 4357 points, the ratio is 0.49918.

Figure 3 illustrates the execution time for computing TIE and TI. For time series with more than 1000 values, the execution time for TIE becomes clearly shorter. For an increasing number of values, the ratio TIE/TE of the executions times decreases.

## 5. Transfer Energy between Polysomnography Time Series Data

One of the earliest applications of the TE presented by Schreiber [6,17] is a study of heart–breath interaction based on TE. The goal of this experiment is to analyze the amount of information transferred between two time series of measurements (heart and breath rates), given the asymmetric character of TE, and to estimate which of the transfer directions (heart → breath or breath → heart) is dominant. In our attempt to reproduce the experiments of Schreiber, we use a box-kernel estimation [6] of the probabilities involved in Equation (3), as suggested by Bossomaier et al. [11].

**Figure 4:**
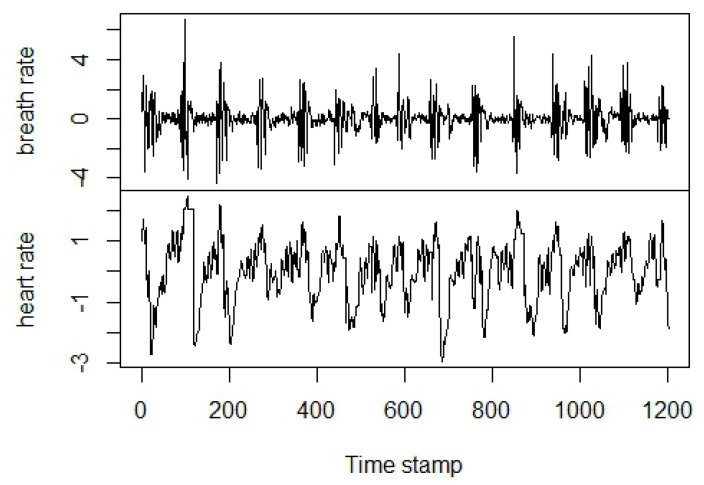
The Santa Fe Data Set B normalized to zero mean and unit variance.

The *MIT-BIH Polysomnographic Database* [27] is a collection of physiological signals recorded during sleep from patients under monitoring for chronic obstructive sleep apnea syndrome. The full version of the dataset is available online (https://www.physionet.org/physiobank/database/santa-fe/) and is offered by PhysioNet [28]. An extract of this dataset known as *Santa Fe Time Series Competition Data Set B* [29] includes heart and breath rates of a patient who shows sleep apnea. To align our methodology with [6], we selected the data points delimited by the indexes 2350–3550, then we normalized the two series to zero mean and unit variance (Figure 4) and evaluated TE and TIE from Equations (3) and (4) with the box-kernel probability estimator [11,30]:

(5) $$p\left(x\right)=\frac{1}{n}\sum_{k=1}^{n}\Theta \left(\frac{x-x_{k}}{r}\right).$$

The kernel function Θ is a similarity measure between the sample points *x* and $x_{k}$ from *X*, whereas *r* is the resolution (or kernel width). When the *step kernel* is selected, that is Θ(|u|≥1)=0 and Θ(|u|<1)=1, we obtain a box-kernel estimator, and p(x) gives the proportion of the points $x_{k}$ falling within the radius *r* around *x*. For *X* representing a random sample of multivariate data, |u| is evaluated as the Euclidean distance between *x* and $x_{k}$, divided by the resolution *r*.

To determine TE and TIE with the Equations (3) and (4), we apply the kernel estimator in Equation (5) to calculate the series of probabilities $p(i_{t})$, $p(i_{t+1},i_{t})$, $p(i_{t},j_{t})$ and $p(i_{t+1},i_{t},j_{t})$ for all instances of $\left[i_{t}\right]$, $\left[i_{t+1},i_{t}\right]$, $\left[i_{t},j_{t}\right]$ and $\left[i_{t+1},i_{t},j_{t}\right]$ obtained from the *Santa Fe Time Series Competition Data Set B*.

We offer now the technical details behind the evaluation of the four series of probabilities mentioned earlier. The first five and the last two instances out of a total of 1201 from the entire dataset are listed in Table 3. In all examples, we denote the *breath rate* values by $\left\{i_{1},i_{2},\cdots ,i_{1201}\right\}$ and the *heart rate* values by $\left\{j_{1},j_{2},\cdots ,j_{1201}\right\}$. We illustrate in the following the computational aspects for r=0.01.

(a) Evaluation of $p(i_{t})$

The probability:

$$\begin{array}{cc} p\left(i_{1}\right)=p\left(0.515\right) & =\frac{1}{1201}\left(\Theta \left(\frac{i_{1}-i_{1}}{r}\right)+\Theta \left(\frac{i_{1}-i_{2}}{r}\right)+\cdots +\Theta \left(\frac{i_{1}-i_{1201}}{r}\right)\right)\\ & =\frac{1}{1201}\left(\Theta \left(\frac{0.515-0.515}{0.01}\right)+\Theta \left(\frac{0.515-1.760}{0.01}\right)+\cdots +\Theta \left(\frac{0.515+1.888}{0.01}\right)\right)\\ & =\frac{1}{1201}\left(1+0+\cdots +0\right)=\frac{5}{1201}=0.00416 \end{array}$$

A similar strategy is applied in the evaluation of the probabilities of all other points $i_{2},\cdots ,i_{1201}$.

(b) Evaluation of $p(i_{t+1},i_{t})$

We take now two consecutive data points from the *breath rate* series and compute the probability of occurrence of one pair of numerical values against all pairs out of a total of 1200 instances:

$$\begin{array}{cc} p(i_{2},i_{1}) & =p(1.760,0.515)\\ & =\frac{1}{1200}\left(\Theta \left(\frac{\left[i_{2},i_{1}\right]-\left[i_{2},i_{1}\right]}{r}\right)+\Theta \left(\frac{\left[i_{2},i_{1}\right]-\left[i_{3},i_{2}\right]}{r}\right)+\cdots +\Theta \left(\frac{\left[i_{2},i_{1}\right]-\left[i_{1201},i_{1200}\right]}{r}\right)\right)\\ & =\frac{1}{1200}\left(\Theta \left(\frac{\left[1.760,0.515]-[1.760,0.515\right]}{0.01}\right)\right.\\ & \left.+\Theta \left(\frac{\left[1.760,0.515]-[1.877,1.760\right]}{0.01}\right)+\cdots +\Theta \left(\frac{\left[1.760,0.515]-[0.041,0.005\right]}{0.01}\right)\right)\\ & =\frac{1}{1200}\left(1+0+\cdots +0\right)=\frac{62}{1200}=0.0516 \end{array}$$

We use the Euclidean distance to compute the difference between two vectors. A similar method is used for the evaluation of all other probabilities $p(i_{t+1},i_{t})$.

(c) Evaluation of $p(i_{t},j_{t})$

In this case, the method is similar to the previous step; the only distinction is $i_{t}$ and $j_{t}$ are picked from the same time stamp, the first one from the *breath rate* series and the second one from the *hearth rate* series.

(d) Evaluation of $p(i_{t+1},i_{t},j_{t})$

The fourth type of probabilities is based on vectors of three components, two consecutive points from the *breath rate* series and one from the *hearth rate* series.

**Figure 5:**
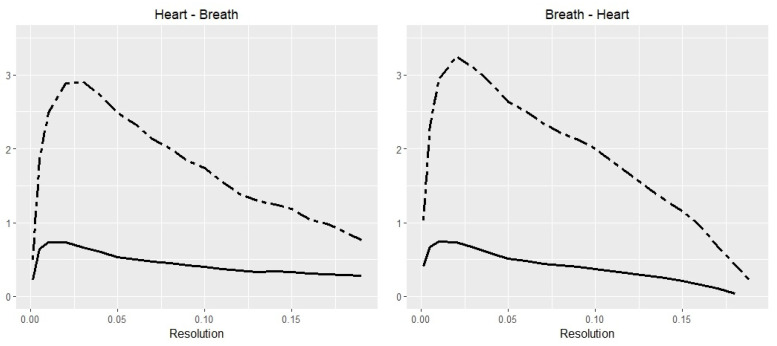
Box-kernel probability estimator for Polysomnography data. The Transfer Entropy (dashed line) from breath to heart has a higher peak than heart to breath. The Transfer Information Energy (solid line) observes a similar pattern, with a correlation coefficient between TE and TIE series of 0.937 for heart → breath and 0.92 for breath → heart.

Figure 5 depicts the TE and TIE values, calculated for resolutions *r* from the interval limited by 0.001 and 0.19 for both directions: heart → breath and breath → heart. The breath → heart direction gives higher values for all resolution points. It is interesting to note that the correlation coefficient between TE and TIE series are 0.937 for heart → breath and 0.92 for breath → heart.

In this application, the time series are short and there is no significant execution time difference for computing TE and TIE. This is in accordance with the results depicted in Figure 3: For time series with more than 1000 values, the execution time for the TIE becomes clearly advantageous. The TIE computation is more scalable than the TE computation.

(e) Discussion about How to Compute the Probabilities

We have to note that, for the discretization of the Financial Time Series (Section 4), the probabilities are computed as relative frequencies with respect to bins, whereas, for the Polysomnography Time Series (Section 5), we used the box-kernel estimation. We did this to illustrate the two different ways for probability estimation.

**Figure 6:**
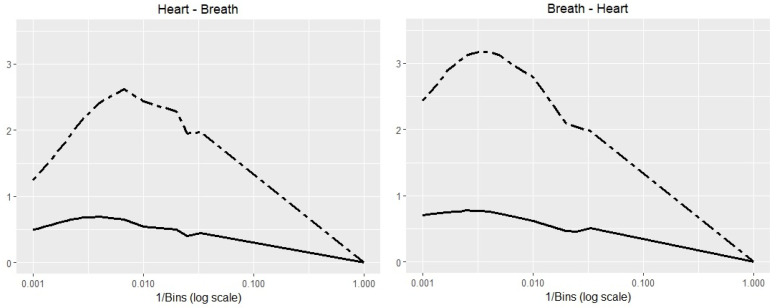
Binned time series estimator for Polysomnography data. The Transfer Entropy (dashed line) from breath to heart has a higher peak than heart to breath. The Transfer Information Energy (solid line) observes a similar pattern. The horizontal axis are represented on a logarithmic scale. The correlation coefficient between TE and TIE series of 0.963 for heart → breath and 0.788 for breath → heart.

A good question is: Are the results independent of the probability estimation approach? In an additional experiment, we estimated the probabilities for the Polysomnography Time Series applications by binning. Then, we computed the TE and TIE which estimate the information transfer in both directions (heart → breath and breath → heart) for various number of bins, ranging 1–1000. The results are depicted in Figure 6. The similarity of the trends is clearly visible. Regardless of the method used in the probability estimation, the results lead to similar conclusions.

We have to note that the approximation accuracy of the bin method is determined by the selected number of bins. The bin method is faster because, by discretization of time series, the number of states is usually much smaller than in the case of the box-kernel method.

## 6. Conclusions

The TE can be used as a measure of the reduction in uncertainty about one time series given another. The TE is already a standard concept: Scheiber’s paper [6] has at this moment 849 citations (https://journals.aps.org/prl/abstract/10.1103/PhysRevLett.85.461).

Symmetrically, the TIE may be viewed as a measure of the increase in certainty about one time series given another. According to our results, the TIE can substitute the TE for measuring information transfer between time series, with the advantage of a computational complexity reduction. We have only compared the effective computation time for the TIE and TE, not the computation time for estimating the probabilities (which is also considerable). However, the same probability estimation is necessary for both the TIE and TE.

For non-Gaussian processes, the TE (and the TIE) and the Granger causality are not measuring the same thing [11]. Since we make no assumptions about Gaussianity, our intention is explicitly to measure information transfer, as opposed to causality in the Granger case. Intentionally, we have not used the term “information flow”, to avoid another controversy (see [31]) related to the the fact that TE measures, or does not measure, the information flow between time series. It is an open problem whether the TIE is an appropriate “energy flow” measure, symmetrical to an information flow.

## Figures and Tables

**Table 1:** The 20 stock market indexes, obtained from the finance.yahoo.com website. We estimate the TE and TIE of all pairs from the 20 stock market symbols. Each symbol represents a time series of daily closing prices recorded during 3 January 2000–14 February 2017.

Americas	Asia/Pacific	Europe
1: MERV	8: AORD	16: ATX
2: BVSP	9: SSEC	17: BFX
3: GSPTSE	10: HSI	18: GDAXI
4: MXX	11: BSESN	19: AEX
5: GSPC	12: JKSE	20: SSMI
6: DJA	13: N255	
7: DJI	14: KS11	
	15: TWII	

**Table 2:** Illustration of the step by step calculation of TE and TIE. Binning the log-returns of the DJI values is subject to slicing the values interval, limited by −0.082 and 0.105. The limits of log-returns of HSI are −0.135 and 0.134. The probabilities are the relative frequencies of symbols or combination of symbols, while TE and TIE can be calculated from the intermediary values ${\mathrm{TE}}_{i}$ and ${\mathrm{TIE}}_{i}$, which are obtained from the probabilities listed on column $t_{i}$.

	$t_{0}$	$t_{1}$	$t_{2}$	$t_{3}$
Closing prices of DJI	11,357.51	10,997.93	11,122.65	11,253.26
Log-returns of DJI		−0.0321	0.0112	0.0116
Binned log-returns of DJI		$i_{1}:g$	$i_{2}:l$	$i_{3}:m$
Closing prices of HSI	17,369.63	17,072.82	15,846.72	15,153.23
Log-returns of HSI		−0.0172	−0.0745	−0.0447
Binned log-returns of HSI		$j_{1}:k$	$j_{2}:f$	$j_{3}:i$
$\left(i_{t+1},i_{t}\right)$		$(i_{2},i_{1}):lg$	$(i_{3},i_{2}):ml$	$(i_{4},i_{3}):nm$
$\left(i_{t},j_{t}\right)$		$(i_{1},j_{1}):gk$	$(i_{2},j_{2}):lf$	$(i_{3},j_{3}):mi$
$\left(i_{t+1},i_{t},j_{t}\right)$		$(i_{2},i_{1},j_{1}):lgk$	$(i_{3},i_{2},j_{2}):mlf$	$(i_{4},i_{3},j_{3}):nmi$
$p(i_{t})$		$p(i_{1})$	$p(i_{2})$	$p(i_{3})$
$p(i_{t+1},i_{t})$		$p(i_{2},i_{1})$	$p(i_{3},i_{2})$	$p(i_{4},i_{3})$
$p(i_{t},j_{t})$		$p(i_{1},j_{1})$	$p(i_{2},j_{2})$	$p(i_{3},j_{3})$
$p(i_{t+1},i_{t},j_{t})$		$p(i_{2},i_{1},j_{1})$	$p(i_{3},i_{2},j_{2})$	$p(i_{4},i_{3},j_{3})$
$\mathrm{TE}=\sum ({\mathrm{TE}}_{t})$		${\mathrm{TE}}_{1}$	${\mathrm{TE}}_{2}$	${\mathrm{TE}}_{3}$
$\mathrm{TIE}=\sum ({\mathrm{TIE}}_{t})$		${\mathrm{TIE}}_{1}$	${\mathrm{TIE}}_{2}$	${\mathrm{TIE}}_{3}$

**Table 3:** The first five instances and the last two out of a total of 1201 pairs of values from the *Santa Fe Time Series Competition Data Set B* normalized to zero mean and unit variance, truncated to three decimal digits.

	Breath Rate	Heart Rate
1	0.515	1.018
2	1.760	1.260
3	1.877	1.514
4	2.913	1.739
5	1.768	1.628
⋯	⋯	⋯
1200	0.005	−1.745
1201	0.041	−1.888
